# Determinants of mortality for adults with cystic fibrosis admitted in Intensive Care Unit: a multicenter study

**DOI:** 10.1186/1465-9921-7-14

**Published:** 2006-01-26

**Authors:** Joëlle Texereau, Dany Jamal, Gérald Choukroun, Pierre-Régis Burgel, Jean-Luc Diehl, Antoine Rabbat, Philippe Loirat, Antoine Parrot, Alexandre Duguet, Joël Coste, Daniel Dusser, Dominique Hubert, Jean-Paul Mira

**Affiliations:** 1 Service de Physiologie, AP-HP, Hôpital Cochin, 27 rue du Faubourg St Jacques, Paris, F-75014, France; 2 Institut Cochin, Département de Biologie Cellulaire, Paris, F-75014 France. Inserm, U567, Paris, F-75014 France. CNRS, UMR 8104, Paris, F-75014 France. Université Paris-Descartes, Faculté de Médecine René Descartes, UMR-S 8104, Paris, F-75014, France; 3 Service de Réanimation Médicale, AP-HP, Hôpital Cochin, 27 rue du Faubourg St Jacques, Paris, F-75014, France; 4 Service de Pneumologie, AP-HP, Hôpital Cochin, 27 rue du Faubourg St Jacques, Paris, F-75014, France; 5 Université Paris-Descartes, Faculté de Médecine René Descartes, UMR-S 8104, Paris, F-75014, France; 6 Service de Réanimation Médicale, AP-HP, Hôpital européen Georges Pompidou, 20 rue Leblanc, Paris, F-75015, France; 7 Service de Pneumologie – Réanimation Médicale, AP-HP, Hôpital Hôtel Dieu, 1 place du Parvis Notre-Dame, Paris, F-75004, France; 8 Service de Réanimation Médicale, Hôpital Foch, 40 rue Worth, Suresnes, F-92150, France; 9 Université Paris VI, Faculté de Médecine Pierre et Marie Curie, Paris, F-75005, France; 10 Service de Réanimation Médicale, AP-HP, Hôpital Tenon, 4 rue de la Chine, Paris, F-75020, France; 11 Service de Pneumologie – Réanimation Médicale, AP-HP, Hôpital Pitié-Salpétrière, 47-83 boulevard de l'Hôpital, Paris, F-75013, France; 12 Service d'Informatique Médicale et de Biostatistique, AP-HP, Hôpital Cochin, 27 rue du Faubourg St Jacques, Paris, F-75014, France

## Abstract

**Background:**

Intensive care unit (ICU) admission of adults with cystic fibrosis (CF) is controversial because of poor outcome. This appraisal needs re-evaluation following recent changes in both CF management and ICU daily practice. Objectives were to determine long-term outcome of adults with CF admitted in ICU and to identify prognostic factors.

**Methods:**

Retrospective multicenter study of 60 ICU hospitalizations for 42 adult CF patients admitted between 2000 and 2003. Reason for ICU admission, ventilatory support provided and one-year survival were recorded. Multiple logistic analysis was used to determine predictors of mortality.

**Results:**

Prior to ICU admission, all patients (mean age 28.1 ± 8 yr) had a severe lung disease (mean FEV_1 _28 ± 12% predicted; mean PaCO_2 _47 ± 9 mmHg). Main reason for ICU hospitalization was pulmonary infective exacerbation (40/60). At admission, noninvasive ventilation was used in 57% of cases and was successful in 67% of patients. Endotracheal intubation was implemented in 19 episodes. Overall ICU mortality rate was 14%. One year after ICU discharge, 10 of the 28 survivors have been lung transplanted. Among recognized markers of CF disease severity, only the annual FEV_1 _loss was associated with a poor outcome (HR = 1.47 [1.18–1.85], p = 0.001). SAPSII (HR = 1.08 [1.03–1.12], p < 0.001) and endotracheal intubation (HR = 16.60 [4.35–63.34], p < 0.001) were identified as strong independent predictors of mortality.

**Conclusion:**

Despite advanced lung disease, adult patients with CF admitted in ICU have high survival rate. Endotracheal intubation is associated with a poor prognosis and should be used as the last alternative. Although efforts have to be made in selecting patients with CF likely to benefit from ICU resources, ICU admission of these patients should be considered.

## Background

Cystic fibrosis (CF) is a common life-shortening genetic disorder among Caucasians caused by mutations of the cystic fibrosis transmembrane conductance regulator gene (*CFTR*), leading to respiratory, pancreatic, and gastro-intestinal disorders [1]. Forty years ago, CF was invariably a fatal disease of early childhood. Although the disease remains incurable, advances in CF chronic disease management (including establishment of specialized care centers, improvement in nutritional care, home ventilatory support and organ transplantation) resulted in increasing the median survival age to 35.1 years [2,3]. In 2003 almost 40% of CF patients were older than 18, corresponding to an adult population with CF of 2,200 individuals in France and 10,000 in the United States illustrating the significant changes in the demographics of CF during the last two decades [3,4]. As severity of CF pulmonary disease usually increases with age, adults are a group at higher risk for acute respiratory complications that are more likely to be life threatening and to request ICU hospitalization [2].

In the 80s, ICU admission of adult patients with CF was restricted since it was associated with a high mortality rate (69% in ICU; 81% at one year), especially when the patients required endotracheal intubation [5]. In the 90s, two studies reported an improved survival rate for this population in ICU. Sood et al. reported an ICU mortality rate of 32% and Vedam et al. showed that 55% of ICU-hospitalized CF patients died in hospital [6,7]. Such differences in outcome may be related to heterogeneity of studied populations and disparities in ICU admission criteria or care. For instance, during the long time periods in which both studies took place (1991–2000 and 1988–2003, respectively), major changes occurred in the respiratory management of exacerbations of chronic cardiopulmonary diseases, including CF. In the last decade, prognosis of acute exacerbations of chronic obstructive pulmonary disease (COPD) dramatically improved due to the increasing implementation of noninvasive ventilation (NIV) [8]. However, time required for the education and training of health careers explains that its daily practice was generalized in ICU only since 1999 [9]. Impact of NIV implementation has been poorly evaluated for CF patients hospitalized in ICU.

Characterizing the adult population recently admitted in ICU and its outcome may help to understand needs and to optimize care for this steadily growing patient group. In this retrospective multicenter study, we have analyzed reasons for ICU admission, initial ventilatory support provided and long-term survival of adult patients with CF. We also determined predictive factors of mortality, which may help to define ICU admission criteria for this population.

## Study population and methods

### Patient selection

Nineteen ICUs of the Paris area (a 10 million people population including approximately 200 adult CF patients) were contacted to identify adult CF patient admissions between January 2000 and June 2003 [4]. Two independent investigators performed report selection by consulting and reviewing ICU clinical databases. Only one episode was considered when patients were transferred from one to another ICU center. CF diagnosis was based on medical history, repeated sweat chloride tests and identification of *CFTR *gene mutations. CF patients who received solid organ transplant before ICU entry were not included in the study. Decisions for ICU admission involved both intensivists and CF clinicians. Patients received standardized care, i.e. antibiotics, nutritional support and chest physiotherapy, according to international guidelines for CF disease [2].

### Data collection

The following data were collected by reviewing patient medical charts: *CFTR *genotype, extra-pulmonary manifestations of CF and features of airway chronic bacterial colonization. The severity of respiratory functional impairment was assessed by using (i) the best baseline lung function test performed during a stable outpatient visit within the 6 months preceding ICU admission, (ii) the slope in lung function decline, calculated from all available lung function tests recorded within the 5 years preceding the entry in the study. Body mass index (BMI) was used as a marker of nutritional state. Home ventilatory support and registration on lung transplant list were recorded as indirect severity indexes.

The slope of lung function decline was calculated individually using linear regression, with a required R^2 ^> 0.40, a minimum follow-up time of two years and a minimum of 4 points for each variable. *CFTR *genotypes were grouped into severity classes according to the probable functional consequences on CFTR protein: "mild" for patients with at least one mutation of classes IV or V; "severe" for patients with two mutations of classes I, II or III; and "not determined" for patients with only one identified mutation of class I, II or III [10].

Demographics, number of previous admissions in ICU, admission source and motive, arterial blood gases and simplified acute physiology score II (SAPSII) were recorded at ICU entry [11]. The need for, the type, the timing and the duration of ventilatory support, the length of ICU stay and patient immediate outcome were also collected.

Six-month and one-year follow-up of patients surviving to ICU hospitalization were obtained from clinicians who usually cared for the patients. In the case of multiple ICU hospitalizations within the study period, the first ICU admission was considered as the index hospitalization.

### Statistical methods

All results are expressed as mean ± SD. Comparisons of the characteristics and outcome of the patients according to ventilatory support were made using nonparametric statistical methods (Wilcoxon's and exact chi-square tests) because of the non-normal distribution of several variables and the small numbers of patients in the groups of interest.

To identify prognostic factors, analyses were based on episodes since several patients underwent re-admissions in ICU during the study period. Cox proportional hazard regression methods were used to determine the association between patient characteristics and outcome. Multilevel modeling was performed to account for the clustering effect of patients (patients having several admissions). Factors that were associated with mortality at *p *value below 0.10 in univariate analyses were considered to enter into the multivariate Cox models. Results are expressed using the hazard ratio (HR), and the 95% confidence interval (CI). STATA software (StataCorp. Stata Statistical Software. Release 7.0, Stata Corporation, College Station-TX-, 2001) was used.

## Results

### Patients

60 admissions corresponding to 42 adult patients with CF (ranging in age from 18 to 54 yr [mean: 28.1 yr]) were identified in 6 medical ICUs of University Hospitals between January 2000 and June 2003. Thirteen ICUs did not admit adult patients with CF during the study period. Among the identified population, 9 patients had more than one admission (maximum five), sometimes in different ICUs. Three patients had experienced ICU care prior to the study period. Mean SAPSII was 21 ± 16 and length of ICU stay was 7.6 ± 7.4 days.

Characteristics of the 42 patients with CF in stable state at the last evaluation before their first ICU admission are shown in Table 1. This population stood out by the severity of the pulmonary disease, characterized by severe bronchial obstruction (FEV_1_: 28 ± 12% predicted value for height, age and sex with a mean slope of annual FEV_1 _loss of 4.21 ± 2.65% predicted/yr, calculated on 4.1 ± 1.4 yr and 12 ± 7 points), chronic hypoxemia and hypercapnia, low body mass index and almost constant chronic airway colonization with *P. aeruginosa *(93%). Before ICU admission, about half of the patients were receiving home ventilatory support (long-term oxygenotherapy or noninvasive ventilation) and one fifth was awaiting lung transplantation.

**Table 1:** Characteristics of the population before the first ICU hospitalization

**Age, yr**	28.1 ± 7.8
**Sex, M/F**	25/17
**Severity of *CFTR *genotype, S/M/ND**	30/4/8
**BMI, Kg/m^2^**	17.8 ± 2.1
	
**Extra-pulmonary involvement**	
Pancreatic insufficiency	37 (88%)
Diabetes	8 (19%)
Cirrhosis	3 (7%)
	
**Chronic airway colonization**	
*P. aeruginosa*	39 (93%)
*B. cepacia complex*	2 (5%)
	
**Lung function in stable state**	
FEV_1_, % predicted	28 ± 12
FVC, % predicted	45 ± 15
TLC, % predicted	98 ± 22
Room air PaO_2_, mmHg	62 ± 15
Room air PaCO_2_, mmHg	47 ± 9
Room air SaO_2_, %	89 ± 8
Annual FEV_1 _loss, % predicted/yr	4.21 ± 2.65
	
**Home ventilatory support**	
Long term oxygenotherapy alone	12 (29%)
Noninvasive ventilation	10 (24%)
	
**Patients awaiting lung transplant**	9 (21%)

Values are expressed in mean ± SD or number (percentage of group).

Abbreviations: ICU: intensive care unit; M: male; F: female; CFTR: cystic fibrosis transmembrane conductance regulator; S: severe; M: mild; ND: not determined; BMI: body mass index; FEV_1_: forced expiratory volume in one second; FVC: forced vital capacity; TLC: total lung capacity; PaO_2_: arterial oxygen tension; PaCO_2_: arterial carbon dioxide tension; SaO_2_: arterial oxygen saturation.

### Admissions

Three respiratory etiologies represented 90% of entry motives in ICU: pulmonary infective exacerbation (n = 40 episodes), moderate to massive hemoptysis (n = 9 episodes) and pneumothorax (n = 5 episodes). Six episodes of hemoptysis required embolization of bronchial arteries and four episodes of pneumothorax needed surgery, underlying the severity of clinical presentation. The other causes for ICU hospitalization were coma (n = 4) (epilepsy, benzodiazepin intoxication) and follow-up after surgical treatment of pneumothorax (n = 2).

ICU admission sources were pulmonary wards (35/60), emergency rooms (20/60), mobile emergency medical units (4/60) and department of surgery (1/60). Sources and reasons for ICU hospitalization of CF patients were tightly associated. While 75% of patients with pulmonary infective exacerbations came from pulmonary wards, admissions for hemoptysis, pneumothorax and other causes were more likely direct (67%, 100% and 50%, respectively).

Last stable spirometric values were significantly higher in patients admitted for hemoptysis (FEV_1_: 35 ± 15%) than in patients hospitalized for pulmonary infective exacerbations (FEV_1_: 25 ± 7%, p = 0.014) or for pneumothorax (FEV_1_: 22 ± 2%, p = 0.025).

### ICU ventilatory support

**Figure 1:**
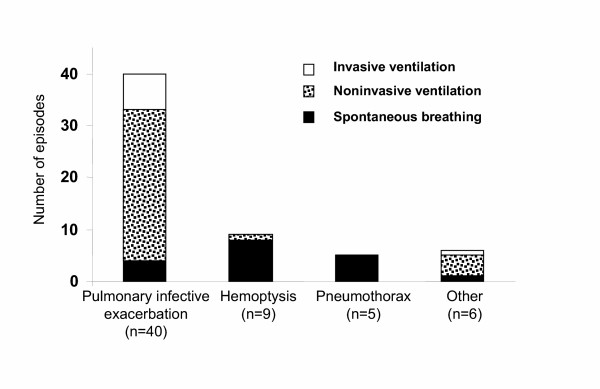
**Initial ventilatory support for the 60 ICU hospitalizations according to reason for admission**. Other causes were coma (epilepsy, n = 2, and benzodiazepin intoxication, n = 2) and follow-up after surgical treatment of pneumothorax (n = 2).

Initial ventilatory assistance was analyzed for the 60 episodes according to admission motives. As illustrated in Figure 1, only one patient required ventilatory support in the group of patients admitted for hemoptysis or pneumothorax.

Noninvasive ventilation (NIV) was the main ventilatory support, initiated at admission in 34 out of the 60 episodes for 7.4 ± 6.5 days. Endotracheal intubation was undertaken before ICU admission in 3 cases, at ICU entry in 5 cases and during ICU stay (at day 3.8 ± 3.9) in 11 cases because of NIV failure. Thus, invasive ventilation (IV) was required in 19 of the 60 episodes for 7.3 ± 8.8 days.

In order to identify factors that may predict the need for and the efficacy of ventilatory support, patients' characteristics were compared according to the initial ventilatory support provided (Table 2) and success or failure of NIV (Table 3). At ICU entry, patients requiring IV had a more severe presentation reflected by higher SAPSII, lower arterial pH and higher PaCO_2_. No features of chronic CF lung disease were predictive for the risk of initial intubation (Table 2). Surprisingly, patients with successful NIV, defined by ICU discharge without endotracheal intubation, had a more severe basal lung function than patients with NIV failure (22 ± 5 vs. 27 ± 8% pred, p = 0.02 for FEV_1_; 52 ± 8 vs. 47 ± 8 mmHg, p = 0.07 for resting PaCO_2_) although no difference appeared in the severity of clinical presentation at ICU entry between both groups (Table 3). Similar findings were found when the successful NIV group was compared to a group composed of both patients with initial IV and patients with NIV failure (data not shown).

**Table 2:** Characteristics and outcome according to initial ventilatory support

	**IV**	**NIV**	**SB**
No. of episodes	8	34	18
Age, yr	29.8 ± 9.2	27.2 ± 8.1	28.9 ± 5.9
Sex, M/F	5/3	20/14	13/5
BMI, Kg/m^2^	17.5 ± 0.8	17.3 ± 1.8	18.2 ± 2.5
			
**Lung function in stable state**			
FEV_1_, % predicted	28 ± 13	24 ± 6	32 ± 14^‡^
Room air PaO_2_, mmHg	67 ± 22	55 ± 12	68 ± 9
Room air PaCO_2_, mmHg	46 ± 10	50 ± 8	44 ± 3^‡^
Annual FEV_1 _loss, % predicted/yr	3.63 ± 3.62	4.33 ± 2.46	3.76 ± 2.46
Home NIV	3 (37%)	18 (53%)	2 (11%)
Waiting for lung transplant	3 (37%)	11 (32%)	1 (6%)
			
**Acute episode characteristics**			
SAPSII	35 ± 28	23 ± 13*	10 ± 4^§^
PaCO_2 _at entry, mmHg	102 ± 60	70 ± 20^†^	49 ± 9^§^
Arterial pH at entry	7.22 ± 0.18	7.36 ± 0.07^†^	7.40 ± 0.05
Admission for pulmonary exacerbation	7 (87%)	29 (85%)	4 (22%)
Length of ICU stay, days	9.6 ± 10.9	8.9 ± 7.7	4.1 ± 3.2
ICU mortality, number of patients (%)	3 (37%)	8 (24%)	0 (0%)

Values are expressed in mean ± SD or number (percentage of group).

Symbols: *: p = 0.067 and †: p < 0.01 for comparison between IV and NIV; ‡: p = 0.005 for comparison between spontaneous breathing and NIV and §: p < 0.001 for comparison between spontaneous breathing and IV or NIV.

Abbreviations: ICU: intensive care unit; NIV: noninvasive ventilation; IV: invasive ventilation; SB: spontaneous breathing; M: male; F: female; BMI: body mass index; FEV_1_: forced expiratory volume in one second; PaO_2_: arterial oxygen tension; PaCO_2_: arterial carbon dioxide tension; SAPSII: simplified acute physiology score II.

**Table 3:** Characteristics and outcome according to NIV effectiveness

	**Success**	**Failure**	
No. of episodes	23	11	p value
Age, yr	26.4 ± 6.9	28.8 ± 10.2	ns
Sex, M/F	15/8	5/6	ns
BMI, Kg/m^2^	17.0 ± 1.5	17.9 ± 2.3	ns
			
**Lung function in stable state**			
FEV_1_, % predicted	22 ± 5	27 ± 8	0.02
Room air PaO_2_, mmHg	54 ± 13	58 ± 11	ns
Room air PaCO_2_, mmHg	52 ± 8	47 ± 8	0.07
Annual FEV_1_ loss, % predicted/yr	4.27 ± 2.55	4.44 ± 2.39	ns
Home NIV	14 (61%)	4 (36%)	0.07
Waiting for lung transplant	8 (35%)	3 (27%)	ns
			
**Acute episode characteristics**			
SAPSII	21 ± 9	28 ± 20	ns
PaO_2 _at entry, mmHg	60 ± 21	66 ± 14	ns
PaCO_2 _at entry, mmHg	70 ± 19	70 ± 24	ns
Arterial pH at entry	7.37 ± 0.06	7.35 ± 0.09	ns
Admission for pulmonary exacerbation	19 (83%)	10 (91%)	ns
Length of NIV, days	9.1 ± 6.8	3.8 ± 3.9	0.02
Length of ICU stay, days	9.1 ± 6.8	8.6 ± 9.5	ns
ICU mortality, number of patients (%)	0 (0%)	8 (73%)	<0.001

Values are expressed in mean ± SD or number (percentage of group). Successful NIV represent episodes requiring NIV use and leading to ICU discharge without endotracheal intubation; NIV failure are episodes requiring both NIV and IV use. Abbreviations: ICU: intensive care unit; NIV: noninvasive ventilation; M: male; F: female; BMI: body mass index; FEV_1_: forced expiratory volume in one second; PaO_2_: arterial oxygen tension; PaCO_2_: arterial carbon dioxide tension; SAPSII: simplified acute physiology score II.

### Outcome

**Figure 2:**
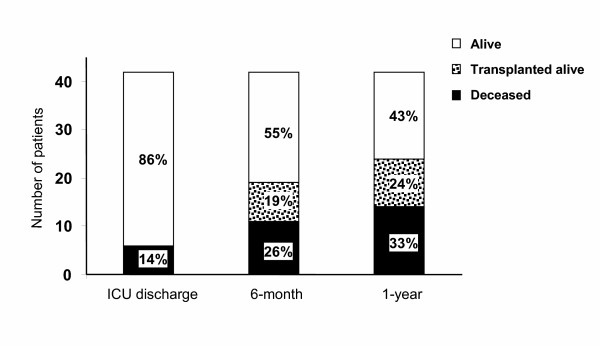
**ICU, six-month and one-year outcome following the first ICU admission**. When a patient was admitted several times, the first hospitalization was used as index hospitalization. Outcome is expressed both in number of patients and percentage of the total population.

Six of the 42 patients (14%) died during their first ICU admission (Fig 2). Mortality rate was 27.5% for patients admitted for pulmonary infective exacerbation while all patients hospitalized for hemoptysis, pneumothorax, post-surgical care or coma survived. Episodes requiring IV had an ICU-mortality rate of 58%. Outcome of patients of the NIV failure group was particularly dramatic, as 8 out of 11 died (Table 3).

One-year follow-up was completed for all patients (Fig 2). Mortality rate increased to 26% at six month and 33% at one year. One-year survival rate was 58% for the subset of patients with pulmonary infective exacerbations. The patients who survived IV were still living at one year; four of them underwent lung transplantation. ICU stay led to a change in chronic management in most survivors, with the implementation of home NIV or home oxygen use in 28% and 25% of the patients, respectively; 8 patients were newly listed for lung transplantation. Among the patients awaiting lung transplantation before the first ICU hospitalization, two died and seven received a graft. The mean time between ICU discharge and lung transplantation (n = 10) was 3.0 ± 3.4 months although patients were on the waiting list for 26.5 ± 24.9 months before ICU hospitalization.

### Predictors of mortality

**Figure 3:**
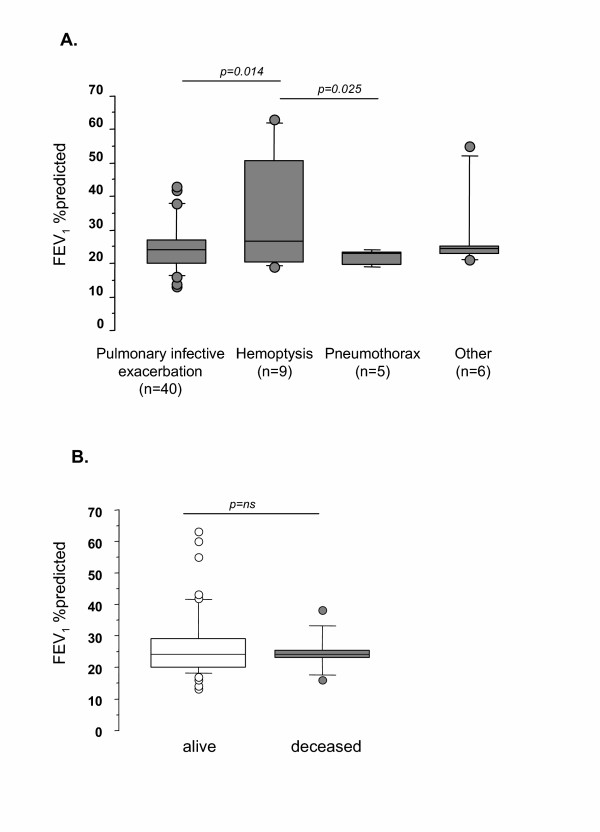
**Lung function parameters in stable state prior to ICU entry according to (A) reason for ICU admission and (B) ICU mortality**. Boxes are interquartile ranges. Bars show range from 10th to 90th percentiles. When a patient was admitted several times, episodes were considered as new and independent events. Other causes were coma (epilepsy, n = 2, and benzodiazepin intoxication, n = 2) and follow-up after surgical treatment of pneumothorax (n = 2). Abbreviations: FEV_1_: forced expiratory volume in one second.

The results of the univariate proportional hazards assessment of risk factors for death are exhibited in Table 4. The need for invasive ventilation was the factor associated with the highest risk of dying (HR = 16.8, 95% CI 4.93 to 57.38, p = 0.001). Among the factors reflecting CF disease severity, a low resting PaO_2 _and an accelerated rate in annual FEV_1 _loss were also associated with an increased risk of death. Age, sex, BMI, severity of *CFTR *genotype, extra-pulmonary involvements, airway chronic bacterial colonization and last stable spirometric data (Fig 3) were not predictive for outcome.

**Table 4:** Univariate analysis of factors associated with mortality

	Hazard Ratio	[95% CI]	p value
**Factors related to severity of CF disease in stable state**			

Age	1.02	[0.91–1.14]	0.701
Sex	1.39	[0.69–2.77]	0.347
BMI	0.95	[0.80–1.13]	0.614
Diabetes	0.79	[0.34–1.85]	0.595
Severity of *CFTR *genotype	0.84	[0.29–2.42]	0.750
*P. aeruginosa *colonization	1.49	[0.40–5.55]	0.544
*B. cepacia complex *colonization	1.95	[0.77–4.89]	0.153
FEV_1_, % predicted	0.97	[0.93–1.02]	0.289
Room air PaO_2_	0.96	[0.93–0.99]	**0.041**
Room air PaCO_2_	1.01	[0.95–1.07]	0.646
Annual FEV_1 _loss, % predicted/yr	1.25	[1.04–1.52]	**0.019**
Long-term oxygenotherapy	1.66	[0.53–5.17]	0.375
Home NIV	1.19	[0.30–4.64]	0.794

**Factors related to ICU hospitalization**			

SAPSII	1.05	[1.02–1.08]	**0.001**
PaO_2 _at entry	1.004	[0.999–1.009]	0.065
PaCO_2 _at entry	1.01	[1.01–1.02]	**0.001**
Initial noninvasive ventilation	12.78	[1.63–100.25]	**0.015**
Initial intubation	14.57	[1.42–149.22]	**0.024**
Intubation during stay	16.82	[4.93–57.38]	**0.001**

Values are expressed as hazard ratios and 95% confidence intervals (CI) for the hazard ratios. When a patient was admitted several times, episodes were analyzed as new and independent events.

Abbreviations: CF: cystic fibrosis; ICU: intensive care unit; BMI: body mass index; CFTR: cystic fibrosis transmembrane conductance regulator; FEV_1_: forced expiratory volume in one second; PaO_2_: arterial oxygen tension; PaCO_2_: arterial carbon dioxide tension; NIV: noninvasive ventilation; SAPSII: simplified acute physiology score II.

The results of the multivariate analysis are presented in Table 5. Three variables, the annual FEV_1 _loss, SAPSII and endotracheal intubation, emerged as significant independent risk factors for death in the final model after adjusting for confounders. Results of the statistical analyses were unchanged when only the patients with pulmonary infectious exacerbations were considered.

**Table 5:** Multivariate analysis for predictors of mortality

**Predictor**	**Hazard Ratio**	**[95% CI]**	**p Value**
Annual FEV_1 _loss	1.47	[1.18–1.85]	0.001
SAPSII	1.08	[1.03–1.12]	<0.001
Endotracheal intubation	16.60	[4.35–63.34]	<0.001

Values are expressed as hazard ratios, 95% confidence intervals (CI) for the hazard ratios and *p*-values. When a patient was admitted several times, episodes were analyzed as new and independent events.

Abbreviations: FEV_1_: forced expiratory volume in one second; SAPSII: simplified acute physiology score II.

## Discussion

Decision to admit adult patients with CF in ICU is still controversial, mostly because of futility reasons. In this multicenter study, we analyzed 60 ICU hospitalizations of 42 adult patients with CF admitted between 2000 and 2003. We have shown that NIV was the main ventilatory support, used in 57% of episodes and 73% of pulmonary infective exacerbations. Despite the pre-existing severe CF lung disease, both ICU and one-year mortality rates were relatively low (14% and 33%, respectively). Severity of acute respiratory failure (reflected by SAPSII and the need for endotracheal intubation) and rapid progression of CF lung disease (illustrated by the yearly decline in percent predicted FEV_1_) were strong independent predictors of mortality.

The increased survival age of patients with CF predicts a shift in patients with life-threatening complications from pediatric ICU to adult ICU [3]. However, effectiveness of ICU admission is still questioned for adult CF patients with advanced lung disease. Since the first report from Davis et al. [5], ICU survival of patients with CF has greatly improved. In the North Carolina hospital series, the retrospective analysis of 106 adult CF patient admissions between 1990 and 1998 (after excluding 30 episodes for antibiotic desensitization) showed that 70% of patients were discharged alive from ICU [6]. A recent study conducted between 1997 and 2001 confirmed these results in a cohort of 23 adult patients with CF (ICU survival rate of 74%) [12]. In the current multicenter report, although patients were older (28 yr old) and harbored more severe lung function than the patients of previous studies, 82% of ICU admissions conducted to ICU discharge. This result is similar to the 17% ICU mortality rate reported in a general ICU population (100,544 patients) hospitalized in the same area in the 90's [13].

This drop in mortality rate may partially result from the significant increase in NIV use in the ICU management of CF patients, despite the absence of evidence-based guidelines. Indeed, this important change in ICU daily practice was essentially based on randomized trials that demonstrated significant reduction in complication and mortality rates for patients with COPD exacerbation [8,14]. Recent evaluation of routine NIV implementation for these COPD patients in ICU showed an increased use from 30% in 1995 to almost 90% in 1999 and a drastic concomitant decrease in in-hospital mortality (from 24% in 1994 to 11% in 2002) [9]. The current study confirms that this new ICU practice also affects respiratory care of adult CF population. Hence, NIV was used in less than 30% of CF patients admitted in the 90's for pulmonary exacerbations and in 60% of cases in the study by Ellafi et al. conducted between 1997 and 2001 [6,7,12]. Here we show that NIV was the main ventilatory support, used in almost 75% of admissions for pulmonary infective exacerbations, and was efficient in two-thirds of the cases. Interestingly, NIV success was more frequent among patients with prior home NIV (Table 3), suggesting the importance of education and chronic respiratory management. An alternative explanation is that these patients were adapted to chronic hypercapnia, hence better suited for worsening hypercapnia than patients with pre-existing normal acid-base status.

NIV failure led to endotracheal intubation and was associated with a very poor outcome (Table 3). The current study showed an overall ICU mortality rate of 58% for patients requiring IV, confirming that prognosis of intubated CF patients has not improved during the last thirty years. Indeed, the study conducted in the 70's and the three ICU-series performed in the 90's concerning either pediatric or adult patients reported an ICU-mortality rate of 65%, 61%, 45% and 61%, respectively, for CF patients requiring endotracheal intubation [5-7,15]. Our results of both univariate and multivariate analyses also strongly highlight the pejorative prognostic value of invasive ventilation (Tables 4 and 5). Conversely, all patients who survived despite IV requirement were still living one year after ICU discharge, half of them having been lung transplanted.

Thus, hospitalization of adult CF patients in ICU may furthermore be justified by both the possibility to adapt chronic management (like implementing home ventilatory support) and the perspective of long-term survival with lung transplantation. In the Sood's study, two thirds of the ICU survivors (20/33) underwent lung transplantation within one year [6]. In our series, 10 of the 28 long-term survivors had received lung transplant, most of them within 6 months after ICU discharge. These results may indicate that CF patients who have been hospitalized in ICU are still good candidates for lung transplants. Furthermore, as previously shown [16], we found that acute episodes requiring IV did not adversely affect the one-year survival benefit after lung transplantation (data not shown).

Severity of CF disease is usually related to several parameters such as *CFTR *genotype, lung function tests, airway bacterial colonization, gender and age [17-20]. Surprisingly, none of these recognized prognostic factors were associated with ICU outcome. Contrasting with previous findings [12,21], no association between *B. cepacia complex *colonization and ICU or long-term survival was found, probably because of the small number of patients infected with this bacteria. Similarly, FEV_1 _value below 30% pred, which serves to consider lung transplantation referral in American and European guidelines [22,23], was not linked to ICU mortality. Only accelerated rate of annual FEV_1 _loss discriminated patients with a higher risk of death although no cut-off value could be determined. Among CF patients, this dynamic variable has been already associated with higher risk of death in a subgroup of patients with severe lung disease (FEV_1 _below 30% pred) [24] but not in a general population heterogeneous for lung function (mean FEV_1 _68% pred) [21]. Differences of studied populations may explain this apparent discrepancy. Hence, rate of decline in lung function is variable along CF pulmonary disease course [25] and it is likely that a rapid FEV_1 _decline may be more accurate to predict mortality in patients with end-stage lung disease. In this particular subgroup of CF patients, occurrence of an acute pulmonary complication might be poorly tolerated. A high-resolution computed tomography study suggested that the loss of FEV_1 _may reflect the severity of bronchial wall thickening and atelectasis-consolidation in CF patients [26]. These features could partially explain the difficulty to reverse severe hypercapnia in some patients despite aggressive ventilatory support.

There are some limits to this study. Thus, we did not consider some important parameters, as they were not contained in medical charts such as patient wishes and quality of life after ICU stay. Because our study relied on observational data, rather than on the results of a prospective trial, the potential for patient selection bias, although small, remains. Nevertheless, patients with CF benefit from regular and standardized medical care in specialized centers that allow precise evaluation of their clinical characteristics at ICU admission and a complete reliable long-term follow-up. The absence of well-defined ICU admission criteria is another important variable to consider for study analysis. However, all ICU admissions analyzed in this study occurred in University Hospitals with Pulmonary Departments that routinely use NIV, suggesting that none of the ICU admission might have resulted from the absence of resources in the pulmonary wards. Analysis of ICU admission sources sustains this statement, as 75% of patients with pulmonary infective exacerbations came from pulmonary wards of the same hospitals. Moreover, comparison of patient characteristics between the 6 ICUs showed no differences in terms of chronic and acute severity of patients (data not shown). A recent French study has shown that CF patients with pulmonary exacerbations admitted in ICU were significantly more severe than those treated in the pulmonary ward of the same hospital [12], underlying that request for ICU admission is mainly triggered by clinical instability, the need for close monitoring or the probability for endotracheal intubation.

## Conclusion

Overall mortality of adult CF patients hospitalized in ICU continues to decrease in recent years, despite admission of older patients with advanced lung disease. Decision to admit these patients in ICU should be considered as surviving to ICU discharge did not compromise opportunity for lung transplantation. Endotracheal intubation was associated with high mortality rate and the use of NIV should be strongly recommended. Parameters used to assess severity of chronic pulmonary disease are poor predictors for ICU outcome and future studies focusing on more relevant markers of CF phenotype are needed to define appropriate ICU admission criteria.

## Abbreviations

BMI: Body Mass Index

CF: Cystic Fibrosis

CFTR: Cystic Fibrosis Transmembrane conductance Regulator

CI: Confidence Intervals

IV: Invasive Ventilation

COPD: Chronic Obstructive Pulmonary Disease

FEV_1_: Forced Expiratory Volume in One second

FVC: Forced Vital Capacity

HR: Hazard Ratio

ICU: Intensive Care Unit

NIV: Noninvasive Ventilation

PaCO_2_: Arterial carbon dioxide tension

PaO_2_: Arterial oxygen tension

SAPSII: Simplified Acute Physiology Score II

SD: Standard Deviation

TLC: Total Lung Capacity

## Competing interests

The author(s) declare that they have no competing interests.

## Authors' contributions

JT participated in the design and coordination of the study and drafted the manuscript.

DJ and GC participated in the design of the study and in data collection in local centers.

JLD, AR, PL, AP and AD participated in acquisition of data.

PRB, DD and DH took part in the interpretation of data and revising.

JC performed the statistical analyses.

JPM conceived the study and revised the draft.

All authors read and approved the final manuscript.

## Appendix

Participating centers for patient follow-up:

Service de Pneumologie (Dr Marc Stern, Dr Dominique Grenet), Hôpital Foch, Suresnes; Service de Pneumologie et Asthmologie Pédiatrique (Pr Pierre Scheinmann, Dr Muriel Le Bourgeois), Hôpital Necker, Paris; Service de Pédiatrie Générale (Pr Gérard Lenoir, Dr Isabelle Sermet), Hôpital Necker, Paris; Service de Pédiatrie (Dr Nicole Hugon), Hôpital Louis Domergue, Trinité; Service de Physiologie Respiratoire-Explorations Fonctionnelles (Pr Josette Dall'ava-Santucci), Hôpital Cochin, Paris.
